# Associations between perceived stress, socioeconomic status, and health-risk behaviour in deprived neighbourhoods in Denmark: a cross-sectional study

**DOI:** 10.1186/s12889-018-5170-x

**Published:** 2018-02-13

**Authors:** Maria Holst Algren, Ola Ekholm, Line Nielsen, Annette Kjær Ersbøll, Carsten Kronborg Bak, Pernille Tanggaard Andersen

**Affiliations:** 1 Unit for Health Promotion Research, Department of Public Health, University of Southern Denmark, Niels Bohrs Vej 9, 6700 Esbjerg, Denmark; 2 National Institute of Public Health, University of Southern Denmark, Øster Farimagsgade 5A, 1353 Copenhagen, Denmark; 3 Unit of Strategy and Analysis, Region of Southern Denmark, Damhaven 12, 7100 Vejle, Denmark

**Keywords:** Health behaviour, Neighbourhood, Deprivation, Socioeconomic status, Perceived stress, Health surveys, Cross-sectional, Regression analysis

## Abstract

****Background**:**

Previous studies have found that residents of deprived neighbourhoods have an increased risk of perceived stress compared to residents with similar sociodemographic and socioeconomic characteristics in non-deprived neighbourhoods. While stress may provide an explanatory pathway linking neighbourhood deprivation to health-risk behaviour, only limited research has been undertaken on whether perceived stress influences health-risk behaviour in deprived neighbourhoods. Moreover, it is uncertain whether perceived stress has a negative effect on the associations between socioeconomic status and health-risk behaviours in deprived neighbourhoods. The overall aim of this study was to compare perceived stress in deprived neighbourhood with that in the general population, and to examine whether perceived stress was associated with health-risk behaviours (including their co-occurrence) in deprived neighbourhoods. A further aim was to examine whether perceived stress modified the associations between socioeconomic status and health-risk behaviours.

****Methods**:**

Four questions from the Perceived Stress Scale were used as indicators of perceived stress. Multiple logistic regression analyses were applied to cross-sectional data from 5113 adults living in 12 deprived neighbourhoods in Denmark. Data from 14,868 individuals from the nationally representative Danish Health and Morbidity Survey 2010 were used as a comparison group with regard to perceived stress.

****Results**:**

Residents of deprived neighbourhoods had higher odds of perceived stress than the general population. Associations between disposable income, economic deprivation, strain, and perceived stress were found in deprived neighbourhoods. Perceived stress was significantly associated with higher odds of health-risk behaviour, including a low intake of fruit or vegetables, daily smoking, physical inactivity, and the co-occurrence of health-risk behaviours, even after adjustment for demographic and socioeconomic characteristics. Perceived stress was more strongly associated with physical inactivity and having two or more health-risk behaviours among residents with medium/high socioeconomic status compared to residents with low socioeconomic status.

****Conclusions**:**

Overall, the study showed a clear association between perceived stress and health-risk behaviour in deprived neighbourhoods. Future health promotion interventions targeting deprived neighbourhoods may benefit from incorporating stress reduction strategies to reduce health-risk behaviour. Further research is needed to fully understand the mechanism underlying the association between perceived stress and health-risk behaviour in deprived neighbourhoods.

**Electronic supplementary material:**

The online version of this article (10.1186/s12889-018-5170-x) contains supplementary material, which is available to authorized users.

## Background

Behaviours such as unhealthy diet, smoking, excessive alcohol intake, and physical inactivity are a major public health issue due to their association with higher risks of morbidity and mortality [1–4]. A strong socioeconomic gradient exists in health-risk behaviour [5, 6], and they are markedly more prevalent among residents of deprived neighbourhoods than among those of non-deprived neighbourhoods [7, 8]. Large differences have been found above and beyond personal characteristics such as sex, age, ethnic background, cohabitation status, educational level, and employment status [7], but the underlying factors that may explain the independent association between neighbourhood deprivation and health-risk behaviours remain poorly understood [7–9]. Studies have suggested that stress could explain the link between neighbourhood deprivation and health-risk behaviours [9–12], thus acting as an aggravating factor that increases health-risk behaviour among residents of deprived neighbourhoods. Residents of deprived neighbourhoods have an increased risk of perceived stress compared to residents with similar sociodemographic characteristics and socioeconomic status (SES) in non-deprived neighbourhoods [9, 13]. Living in deprived neighbourhoods may lead to increased stress through factors such as overcrowding, high crime rates, perceived danger, poor transportation, poor housing, disrepair, limited services, poor infrastructure, and a lack of social support [13–18]. Additionally, neighbourhood deprivation is an independent source of stress, over and above individual SES and other factors [17]. Stress is associated with health-risk behaviour [19–27], possibly in large part because people often cope with feelings of stress through risky, but often pleasurable, behaviours [10, 20, 21, 28–33] such as eating high-fat foods, smoking, and drinking alcohol [10]. Furthermore, the motivation for physical activity may be limited when experiencing stress [10].

There is only limited research into whether perceived stress can influence health-risk behaviour among residents of deprived neighbourhoods. A study of women in deprived neighbourhoods in Australia found cross-sectional and longitudinal associations between perceived stress and both reduced leisure-time physical activity and more frequent fast food consumption [34]. Further research into perceived stress and health-related behaviours in deprived neighbourhoods is warranted, including comparisons with the general population [18]. There appear to be large socioeconomic differences in health-risk behaviours among residents in deprived neighbourhoods [7], and it seems plausible that perceived stress can increase socioeconomic differences in health-risk behaviour. Whether perceived stress has an aggravating effect on the associations between SES and health-risk behaviour in deprived neighbourhood remains uncertain, as no published studies have examined this. A better understanding of health-risk behaviours in the context of stress and coping may be useful for health promotion interventions aimed at reducing health-risk behaviour in deprived neighbourhoods and may reduce social inequality in health in general.

The aim of this study was threefold: First, to compare the prevalence of perceived stress among residents in deprived neighbourhood with that in the general population. Second, to determine whether perceived stress was associated with health-risk behaviour (including their co-occurrence) among residents of deprived neighbourhoods. Third, to examine whether the association between SES and health-risk behaviour among residents of deprived neighbourhoods was modified by perceived stress.

## Methods

### Data materials

#### Deprived Neighbourhood health profile survey

Data from the cross-sectional survey *Deprived Neighbourhood Health Profile Survey* (DNHPS) were provided by the Danish Health Authority. DNHPS was collected in 12 deprived neighbourhoods in Denmark during January–March 2011 as part of a government-funded health intervention project [35]. The Danish Health Authority based their selection of deprived neighbourhoods on a number of criteria at municipality level [36]. For example, the municipalities had to show evidence of the need and potential for health interventions in a geographically bounded neighbourhood with a high proportion of less resourceful residents (for example, people on transfer income or with poor connection to the labour market). However, no specific criteria for demographic or socioeconomic characteristics were specified [7].

The DNHPS was based on a stratified random sample of 8835 households, and residents aged 18 years or older were invited to participate in the survey. A total of 5113 interviews were collected (response rate: 63%). Further information for each neighbourhood on the number of households, number of residents aged 18 or older in the neighbourhood, gross sample, and number of completed interviews are shown elsewhere [7]. The survey was conducted through telephone interviews supplemented with face-to-face interviews to increase the response rate among residents with non-Western background. The survey is described in detail elsewhere [7].

#### General population: Danish health and morbidity survey 2010

Data on the general population were obtained from the *Danish Health and Morbidity Survey 2010* (DHMS) [37], which randomly sampled 25,000 Danes aged 16 years or above, selected from the Danish Civil Registration System (each Danish citizen is given a unique personal registration number at birth or immigration). The survey was conducted in February–April 2010 by the National Institute of Public Health, and 15,165 individuals completed the self-administered questionnaire (response rate: 61%) [37]. For the purposes of the current study, we included data from persons aged 18 years or above (*n* = 14,686), reflecting the age distribution of the residents of the deprived neighbourhoods. The survey is described in more detail elsewhere [37].

### Variables

#### Perceived stress

Perceived stress was measured using four questions from the Perceived Stress Scale (PSS) that describes life stress in terms of feeling in control [38]. The specific questions were: 1) “In the last month, how often have you felt nervous or stressed?”, 2) “In the last month, how often have you felt that you could not cope with all the things that you had to do?”, 3) “In the last month, how often have you felt difficulties were piling up so high that you could not overcome them?”, and 4) “In the last month, how often have you felt that you were on top of things?” The response categories were *Never*, *Almost never*, *Sometimes*, *Fairly often*, and *Very often*. The second DHMS question was slightly modified in the DNHPS into: “In the last month, how often have you felt that you could not cope with the things you would like to achieve in your daily life?”

Responses to the three first questions were dichotomised into (a) *Very often/Often* and (b) *Never/Almost never/Sometimes*, with (a) indicating a high level of stress. Responses to the last question were dichotomised into (a) *Never/Almost never* and (b) *Sometimes/Often/Very often*, with (a) indicating a high level of stress.

An index of perceived stress was constructed by summing the responses to the four questions. Perceived stress was defined as the presence of one or more indicators of high level of stress. The index of perceived stress had a Cronbach’s alpha coefficient of 0.79, which implies good internal consistency (online Additional file 1: Table S1).

#### Health-risk behaviour

Health-risk behaviour was measured using four indicators: low intake of fruit or vegetables, daily smoking, high-risk alcohol intake, and physical inactivity. A low fruit or vegetable intake was defined as respondents who did not eat fruit or vegetables every week and was categorized as “Low intake of fruit or vegetables”*.* Daily smoking was defined as respondents who reported that they currently smoked every day. Alcohol intake was based on the self-reported number of standard alcohol drinks consumed during a typical week. The Danish Health Authority’s definition of high-risk alcohol intake was applied (> 14 standard drinks per week for women; > 21 standard drinks per week for men) [39]. Physical inactivity was assessed by one question about the respondents’ typical level of physical activity in their leisure time during the past 12 months, using four predefined response categories: *Heavy exercise and competitive sports regularly and several times a week*; *Exercise or heavy gardening at least four hours a week*; *Walking, biking or other light exercise at least four hours a week (including Sunday excursions, light gardening and cycling or walking to work)*; and *Reading, watching TV or other sedentary activity*. The latter category was used to define physical inactivity.

#### The co-occurrence of health-risk behaviours

Information on the co-occurrence of health-risk behaviours was based on a risk factor score calculated by summing the respondent’s health-risk behaviours in relation to fruit and vegetable consumption, smoking, alcohol intake, and physical activity. The following four health-risk behaviours were chosen: low intake of fruit or vegetables, daily smoking, high-risk alcohol intake, and physical inactivity.

#### Sociodemographic and socioeconomic characteristics

Measures of sociodemographic and socioeconomic characteristics included sex, age, ethnic background, educational level, employment status, and cohabitation status. In the DNHPS, all sociodemographic and socioeconomic characteristics were self-reported. In the DHMS, information on the respondents’ sex and age was extracted from the Danish Civil Registration System [40], which was also the source of data used to determine ethnic background. Ethnic background was categorized into three groups: Danish background, other Western background (from the 28 European Union member states and Andorra, Iceland, Liechtenstein, Monaco, Norway, San Marino, Switzerland, Vatican City, Canada, the USA, Australia, and New Zealand) or non-Western background (all other countries). Data on highest level of education completed and employment status were self-reported. Education was categorised according to Statistics Denmark’s definitions, ranging from “No education/Basic school” to “Long-cycle higher education” [41]. An SES index was constructed based on educational level and employment status and was dichotomised into (a) Low and (b) Medium/high SES, where the former category included respondents who had not studied beyond primary school and were not employed.

#### Disposable income, economic deprivation, and strain

The median disposable income after fixed expenses among the residents of the deprived neighbourhoods was calculated to be DKK 4000 per month (approximately USD 700/GBP 450, at 2011 currency rates of exchange) [42]. This was used as a cut-off point for comparing worse-off and better-off residents of the deprived neighbourhoods. Monthly disposable income was dichotomised into (a) Low disposable income (< DKK 4000) and (b) High disposable income (≥ DKK 4000). Economic deprivation was based on whether the respondent had to refrain from doing one or more of the following activities: leisure activities, giving gifts for birthdays or other occasions, going to the dentist, buying essential medicines, buying clothes or shoes for sport or exercise, and incidental expenses. The economic deprivation variable was dichotomised into (a) Suffering from economic deprivation and (b) Not suffering from economic deprivation. Strain was measured using a question about whether the respondent had been strained within the past year by finances, housing situation, work situation, relationship with their partner or children, illness in the family or among close friends, etc. Strain was dichotomised into (a) Having one or more strains, and (b) No strains.

It was not possible to examine disposable income, economic deprivation, and strain in the general population, as these items were not included in the DHMS.

### Statistical analysis

As a first step, exploratory factor analysis (EFA) was used to explore whether it was possible to construct an index for perceived stress using varimax rotation (orthogonal). A complete case analysis was performed due to the missing data values. As the results were the same for all cases and complete cases the missing values could not have affected the results.

Multiple logistic regression analyses were used to compare perceived stress among residents of the deprived neighbourhood with that in the general population. The results are presented as odds ratios (OR) with 95% confidence intervals (95% CI). The logistic regression model was adjusted for sex, age, ethnic background, educational level, cohabitation status, and employment status, as these are important determinants of health behaviour [37] (the same adjustment strategy was applied in all regression models in the present study). When the models were adjusted for educational level, the analyses were restricted to individuals aged 25 years or older as they were assumed to have completed their education. When the model was adjusted for employment status, the analyses were restricted to respondents aged 25–64 years and to employed, unemployed, disability pensioners, and other non-employed individuals (including home-makers, people on long-term sick leave, rehabilitated, benefit claimants, and non-classifiable people). These restrictions were applied for all regression models including educational level and employment status. Multiple logistic regression analyses were also used to investigate the associations between disposable income, economic deprivation, and strain, with perceived stress as the outcome.

Multiple logistic regression analyses were also applied to investigate associations between perceived stress and various health-risk behaviour outcomes among the residents of the deprived neighbourhoods, and to investigate whether perceived stress modified the associations between SES and health-risk behaviour among residents of deprived neighbourhoods.

The DNHPS questionnaire offered a “Do not know” category, which was treated as missing in the analyses. In any case, these involved a negligible number of responses across the variables (< 2.7% of cases). Statistics Denmark computed calibrated weights to reduce the impact of non-response bias on the estimates in the DHMS. Register information on sex, age, ethnic background, educational level, and income, etc., for all persons invited to participate in the DHMS was obtained to calculate the weights [37]. Thus, persons in underrepresented groups were given a higher weight (a weight greater than 1) in the analyses to represent the larger number of non-respondents with similar characteristics. Persons in overrepresented groups were given a weight less than 1.

All analyses were carried out using SAS version 9.3.

## Results

### Comparison with the general population

The prevalence of perceived stress was 33.6% for residents of deprived neighbourhoods and 26.7% in the general population (Table 1). When the model was adjusted for sex, age, and ethnic background, residents of deprived neighbourhoods had 1.30 times higher odds (95% CI: 1.21–1.40) of perceived stress than the general population. Significant associations were observed even after further adjustment for educational level, cohabitation status, and employment status (see Additional file 2: Table S2).

**Table 1:** Sociodemographic and socioeconomic characteristics, and proportions with perceived stress in the deprived neighbourhoods and the general population

	Study population, % (n)				Proportion with perceived stress, %	
	Deprived neighbourhoods		General population		Deprived neighbourhoods	General population
Total	5113		14,686		33.6	26.7
Sex						
Men	45.8	2342	45.8	6731	30.3	22.9
Women	54.2	2771	54.2	7955	36.5	30.5
Age (years)						
18–24	9.0	460	8.1	1192	39.6	31.6
25–44	33.9	1734	28.6	4197	40.3	25.3
45–64	35.0	1791	39.1	5743	33.6	24.4
≥ 65	22.0	1126	24.2	3554	20.9	30.8
Ethnic background						
Danish	82.8	4235	94.1	13,812	31.3	25.5
Western	2.1	106	2.7	396	34.0	32.9
Non-Western	15.1	772	3.3	478	47.4	42.5
Cohabitation status						
Cohabiting	44.7	2287	73.5	10,531	30.4	23.6
Living alone	55.3	2826	26.5	3798	36.3	32.4
Highest educational level						
No education/Basic school	32.6	1664	11.4	1624	35.6	37.9
Upper secondary or vocational school	36.0	1837	34.7	4929	31.7	25.5
Short-cycle higher education	10.0	508	11.7	1659	34.7	24.1
Medium-cycle higher education	11.0	610	19.5	2773	30.7	21.0
Long-cycle higher education	3.5	179	11.1	1585	29.3	19.4
Other education^a^	6.0	307	11.6	1656	41.5	34.7
Employment status						
Employed	41.3	2108	57.3	8057	28.8	20.5
Unemployed	14.7	752	3.1	440	51.7	43.7
Disability pensioner	9.9	504	3.8	529	49.5	57.9
Other non-employed^b^	34.1	1743	35.8	5043	27.3	31.1
Socioeconomic status (SES)^1^						
Low SES	23.3	1191	9.8	1414	38.0	42.6
Medium/high SES	76.7	3920	90.2	13,095	32.4	25.0

^a^Including “Still attending school”

^b^Others (E.g. student, early retirement/age pensioners)

^1^Based on highest educational level and employment status

### Sociodemographic and socioeconomic characteristics

The prevalence of perceived stress was highest among the unemployed and disability pensioners, irrespective of whether they lived in the deprived neighbourhoods or were among the general population. Up to the age of 65, the prevalence of perceived stress was higher among residents of deprived neighbourhoods than in the general population, but it was substantially more prevalent in the oldest age group in the general population. (Additional file 3: Table: S3) shows the prevalence of the items used to construct the perceived stress index.

### Disposable income, economic deprivation, and strain

Table 2 shows prevalence and ORs (unadjusted and adjusted) of the association between perceived stress and disposable income, economic deprivation, and strain among residents of deprived neighbourhoods. Additional file 4 shows the prevalence of all response categories for disposable income (Table S4), economic deprivation (Table S5), and strain (Table S6). The adjusted ORs of perceived stress were significantly higher among residents with a low monthly disposable income (OR: 1.57; 95% CI: 1.35–1.83) compared to those with a high monthly disposable income. Economic deprivation was significantly associated with higher odds of perceived stress (OR: 2.90; 95% CI: 2.53–3.33). The odds of perceived stress increased with the number of strain factors (OR: 1.79; 95% CI: 1.71–1.88). Further analysis showed that residents experiencing at least one strain had 5.29 times higher odds (95% CI: 4.31–6.50) of perceived stress than residents with no strains.

**Table 2:** Associations between perceived stress and disposable income, economic deprivation and strain in deprived neighbourhoods. ORs with 95% CI for perceived stress

		Unadjusted	Adjusted		
	%	OR 95% CI	OR^a^ 95% CI	OR^b^ 95% CI	OR^c^ 95% CI
Disposable income					
High disposable income	28.9	1.00	1.00	1.00	1.00
Low disposable income	43.8	**1.92 (1.69–2.19)**	**1.95 (1.71–2.23)**	**1.78 (1.54–2.06)**	**1.57 (1.35–1.83)**
Economic deprivation					
Not suffering from economic deprivation	22.4	1.00	1.00	1.00	1.00
Suffering from economic deprivation	52.3	**3.81 (3.36–4.31)**	**3.37 (2.96–3.84)**	**3.2 (2.82–3.69)**	**2.90 (2.53–3.33)**
Strain					
Having strains		**1.84 (1.76–1.92)**	**1.80 (1.72–1.88)**	**1.78 (1.70–1.86)**	**1.79 (1.71–1.88)**
Having no strains	9.7	1.00	1.00	1.00	1.00
Having one or more strains	41.5	**6.60 (5.40–8.06)**	**5.89 (4.81–7.21)**	**5.84 (4.77–7.16)**	**5.29 (4.31–6.50)**

Bold values indicate significant odds ratios

^a^Adjusted for sex, age and ethnic background

^b^Adjusted for sex, age, ethnic background, educational level and cohabitation status. Analysis restricted to respondents aged 25 years or older

^c^Adjusted for sex, age, ethnic background, educational level, cohabitation status and employment status. Analysis restricted to respondents aged 25–64 years and employed, unemployed, disability pensioners and other non-employed

### Health-risk behaviour

Table 3 shows the association between perceived stress and health-risk behaviour among residents of deprived neighbourhoods. When the analyses were adjusted for sex, age, ethnic background, educational level, and cohabitation status, the experience of perceived stress was significantly associated with higher odds of health-risk behaviour, including low fruit or vegetable intake (OR: 1.56; 95%: 1.22–1.99), daily smoking (OR: 1.59; 95% CI: 1.39–1.82), physical inactivity (OR: 1.95; 95% CI: 1.67–2.28), and co-occurrence of health-risk behaviours. When the analyses were additionally adjusted for employment status, the result for low fruit and vegetable intake was no longer significant. No significant association between perceived stress and high-risk alcohol intake was found. Interaction analyses showed no significant association between sex and perceived stress with regard to health-risk behaviour (all *p*-values > 0.05).

**Table 3:** Associations between perceived stress and health-risk behaviours in deprived neighbourhoods. ORs with 95% CI for health-risk behaviours

	%		Unadjusted	Adjusted		
	Perceived stress	No perceived stress	OR 95% CI	OR^a^ 95% CI	OR^b^ 95% CI	OR^c^ 95% CI
Fruit and vegetables						
Low intake of fruit or vegetables	10.1	6.4	**1.63 (1.32–2.01)**	**1.83 (1.47–2.28)**	**1.56 (1.22–1.99)**	1.25 (0.94–1.68)
Smoking						
Daily smoking	46.6	33.9	**1.71 (1.51–1.92)**	**1.71 (1.51–1.93)**	**1.59 (1.39–1.82)**	**1.49 (1.27–1.75)**
Alcohol						
High-risk alcohol intake	5.9	5.5	1.07 (0.83–1.38)	1.27 (0.98–1.64)	1.19 (0.90–1.57)	1.20 (0.86–1.67)
Physical activity						
Physical inactivity	25.1	15.6	**1.81 (1.56–2.09)**	**1.91 (1.65–2.22)**	**1.95 (1.67–2.28)**	**1.56 (1.29–1.90)**
Co-occurrence of health-risk behaviours						
Having 2 or more health-risk behaviours	20.9	12.3	**1.89 (1.61–2.21)**	**2.03 (1.73–2.39)**	**1.93 (1.62–2.30)**	**1.59 (1.28–1.96)**
Having 3 or more health-risk behaviours	5.1	2.4	**2.17 (1.59–2.97)**	**2.57 (1.86–3.55)**	**2.10 (1.48–2.98)**	**1.57 (1.03–2.38)**

Bold values indicate significant odds ratios

^a^Adjusted for sex, age and ethnic background

^b^Adjusted for sex, age, ethnic background, educational level and cohabitation status. Analysis restricted to respondents aged 25 years or older

^c^Adjusted for sex, age, ethnic background, educational level, cohabitation status and employment status. Analysis restricted to respondents aged 25–64 years and employed, unemployed, disability pensioners and other non-employed

### Perceived stress as an effect modifier

Table 4 shows health-risk behaviour by combinations of SES and perceived stress in deprived neighbourhoods. The combined variable included four possible combinations of SES and perceived stress: Low SES and perceived stress (*n* = 251), Low SES and no perceived stress (*n* = 248), Medium/high SES and perceived stress (*n* = 875), and Medium/high SES and no perceived stress (*n* = 1669). The combination of low SES and perceived stress showed higher odds of health-risk behaviour (except for high-risk alcohol intake) and co-occurrence of health-risk behaviour than the combination of medium/high SES and no perceived stress. Residents with low SES and perceived stress had higher odds of all health-risk behaviours (except for high-risk alcohol intake) and co-occurrence of health-risk behaviour than residents with higher SES and no perceived stress. Perceived stress modified the associations between SES and physical inactivity (*p* = 0.0227) and between SES and having two or more health-risk behaviours (*p* = 0.0275). The modification analyses showed that perceived stress was strongly associated with physical inactivity and having two or more health-risk behaviours among residents with medium/high SES compared to residents with low SES. Moreover, the analysis showed a significant interaction between sex and the combination of SES/perceived stress with regard to exhibiting three or more health-risk behaviours (*p*-value = 0.0203).

**Table 4:** Health-risk behaviours by combinations of socioeconomic status (SES) and perceived stress in deprived neighbourhoods. Adjusted ORs with 95% CI for health-risk behaviours

OR^a^ (95% CI)						
	Low intake of fruit or vegetables	Daily smoker	High-risk alcohol intake	Physical inactivity	Having 2 or more health-risk behaviours	Having 3 or more health-risk behaviours
Combined variable of SES and stress	*p* = 0.6103*	*p* = 0.3293*	*p* = 0.6430*	***p*** = 0.0227*	***p*** = 0.0275*	*p* = 0.3178
Low SES and perceived stress	**3.10 (2.02–4.78)**	**3.44 (2.79–4.23)**	1.38 (0.77–2.48)	**2.47 (1.80–3.38)**	**3.17 (2.27–4.43)**	**2.06 (1.04–4.10)**
Low SES and no perceived stress	**2.50 (1.62–3.86)**	**1.99 (1.52–2.62)**	1.21 (0.69–2.14)	**2.19 (1.59–3.02)**	**2.66 (1.91–3.71)**	1.61 (0.80–3.25)
Medium/high SES and perceived stress	**1.46 (1.04–2.04)**	**1.69 (1.42–2.00)**	1.39 (0.96–1.98)	**1.90 (1.53–2.35)**	**2.03 (1.61–2.56)**	**2.10 (1.33–3.32)**
Medium/high SES and no perceived stress	1.00	1.00	1.00	1.00	1.00	1.00

Bold values indicate significant odds ratios

^a^Adjusted for sex, age, ethnic background and cohabitation status. Analysis restricted to respondents aged 25–64 years and employed, unemployed, disability pensioners and other non-employed

^*^P-value for interaction between SES and perceived stress in regard to each health-risk behaviour. Bold values indicate significant interactions

## Discussion

This study examined associations between perceived stress, SES, and health-risk behaviours among residents of deprived neighbourhoods, and whether perceived stress modified the associations between SES and health-risk behaviours. Consistent with prior research, we found a significantly higher risk of perceived stress among residents of deprived neighbourhoods compared with the general population [9, 13]. The study found that perceived stress in deprived neighbourhoods was significantly associated with low intake of fruit or vegetables, daily smoking, physical inactivity, and the co-occurrence of health-risk behaviours. However, perceived stress was not associated with high-risk alcohol intake. Additionally, perceived stress modified the associations between SES and physical inactivity and between SES and having two or more health-risk behaviours in the deprived neighbourhoods. Perceived stress was strongly associated with physical inactivity and having two or more health-risk behaviours among residents with medium/high SES compared to residents with low SES.

The findings of this study support similar studies of associations between stress and health-risk behaviour in other populations [19–27]. The association between stress and low fruit or vegetable intake has also been found by prior research [23, 43]. In a study by Mouchacca et al., stress was found to predict higher fast food consumption among women from deprived neighbourhoods in Australia [34]. In addition, a systematic review reported less healthy eating patterns among people in lower social positions who had higher stress levels [24]. Unhealthier eating habits of people with higher levels of stress may be due to unhealthy foods being perceived as a ‘comfort’ or ‘reward’ in coping with stress [44].

Our findings are in line with several studies showing that high levels of perceived stress are associated with increased smoking levels, smoking initiation, and a reduced likelihood of quitting smoking [19–21, 25–27, 45]. Among others, Kaplan et al. found that smokers with high levels of perceived stress tended to smoke more and were less confident that they would be able to quit smoking [45]. There is strong evidence that cigarette smoking can be a coping mechanism that provides a respite from stressful physical environments such as overcrowding, low-quality housing, traffic, and neighbourhood noise [46, 47].

The absence of an association between perceived stress and high-risk alcohol intake in deprived neighbourhoods is supported by Ng and Jeffery’s study, in which no association between high perceived stress levels and the level of alcohol intake was found [20]. Other studies have, however, reported a positive association between stress and high-risk alcohol intake [25–27, 48]. These inconsistent results could be due to the different methods used to measure stress and alcohol intake or differences in the studied populations. It is worth mentioning that the correlation between socioeconomic factors and alcohol intake in Denmark is very modest when compared to other Western countries [49]. In Denmark, high-risk alcohol intake is more prevalent in younger people with basic school as their highest level of education, whereas the most risk-prone in older age groups are those with the highest education [50]. The Danish alcohol culture can also be characterised as relatively ‘wet’, where drinking is a fundamental factor of social life for many in the general population [51]. However, this is not the case in deprived neighbourhoods [7].

The association between perceived stress and physical inactivity among residents of deprived neighbourhoods was unsurprising, as previous studies have reported that stress is associated with reduced physical activity and increased sedentary behaviours [19, 20, 26, 27]. However, Steptoe et al. found no differences in exercise frequency or duration with changes in perceived stress [48]. Reduced participation in physical activity can be caused by stress due to difficult life circumstances and lack of coping, which may go beyond self-care and health-promoting behaviours such as physical activity [26]. In addition, many people respond to stress by engaging in less physical activity because they may consider sedentary physical activity rewarding in the short term, despite evidence that physical activity can reduce stress over time [20, 26, 52, 53].

Our finding on the association between perceived stress and the co-occurrence of health-risk behaviours is in line with Fine et al., who reported that persons with high mental distress are twice as likely to have three or four health-risk behaviours than none or one health-risk behaviour [54]. Our findings highlight that deprived neighbourhood residents with perceived stress constitute a risk group for the co-occurrence of health-risk behaviours.

In the research on the association between stress and health-risk behaviour, there is often the perception that health-risk behaviour can be a tool to cope with stress [10, 20, 21, 28–33] because behaviours such as smoking and eating high-fat food give immediate pleasure [20, 33]. However, research cannot establish with certainty that health-risk behaviours are used as coping tools for stress [30]. Our results showing higher odds for health-risk behaviour among residents with perceived stress offer indirect support for health-risk behaviour as a coping tool. In Kaplan et al.’s focus group study of residents in low-income communities, the participants described a direct causal pathway between stress and poor health as well as an indirect pathway through health behaviours, including uncontrolled eating, smoking, and physical inactivity [45]. The study participants articulated various theories about the links between stress and health-risk behaviour, such as self-medication, adaptive behaviour, discounting the future, depletion of willpower, and competing priorities [45].

Our study also found that disposable income, economic deprivation, and strain had strong associations with perceived stress, which may partly explain why residents of deprived neighbourhoods have a higher risk of perceived stress than the general population. Wilkinson has suggested that the poor suffer doubly from deprivation: besides the direct material effects, it also affects their health through psychosocial channels [55]. Being at the bottom of society’s hierarchy may lead to stress from feelings of bitterness based on invidious social comparisons [56], and the perception of social inequality can be an incentive for health-risk behaviour [28]. In the same way, living in a neighbourhood which is deprived not only in absolute terms, but also relative to nearby neighbourhoods and to society in general, can induce feelings of exclusion and stigmatisation, and residents in deprived neighbourhoods may resort to health-risk behaviours to cope with these perceptions [57].

The findings of this study have important implications for public health practice. Health promotion interventions would benefit from incorporating stress reduction strategies to address health-risk behaviour in deprived neighbourhoods. We recommend interventions to help residents of deprived neighbourhoods to cope with stress without resorting to health-risk behaviour—especially in regard to fruit and vegetable consumption, smoking, and physical activity. These interventions should help residents to acknowledge the short-term nature of the rewards of health-risk behaviours and to find other more effective and less harmful ways of coping with stress. Previous research has found that activities such as physical activity, relaxation techniques, talking to others, or making time for social activities were effective in managing stress [30, 58]. Lipschitz et al. found that persons with poor stress management had more health-risk behaviours than those with effective stress management [59]. The study also found a relationship between improved stress management over six months and decreasing health-risk behaviour [58, 59]. Furthermore, as mental health has shown to be associated with health behaviours, we recommend the adoption of general mental health promotion initiatives in deprived neighbourhoods [60]. Practitioners should assess perceived stress and refer their patients to stress reduction facilities when appropriate. Knowledge about perceived stress among residents of deprived neighbourhoods can be used to identify vulnerable groups who need special attention in health promotion interventions aimed at deprived neighbourhoods.

The idea that perceived stress caused by neighbourhood deprivation can increase the risk of health-risk behaviour arises mainly from cross-sectional studies associating neighbourhood deprivation with perceived stress [10, 13]. The results of this study provide a base for future longitudinal studies to examine causal associations between perceived stress and health-risk behaviour in deprived neighbourhoods.

Future research should also examine pathways between perceived stress and health-risk behaviour in deprived neighbourhoods to gain a deeper understanding of the mechanisms through which perceived stress causes health-risk behaviour. This should include identifying of modifiers and mediators of this association, which could provide important target areas for future interventions. The influence of perceived stress on health-risk behaviours may depend, for example, on the availability of buffering resources and stress-modifying factors such as social support or personality characteristics [61, 62]. In general, socioeconomically deprived groups have fewer buffering resources, making them more prone to stress and therefore more likely to cope via health-risk behaviour than higher socioeconomic groups [61–63]. Further research into interpersonal and intrapersonal coping resources for stress in deprived neighbourhoods would be useful, e.g. on social networks that can provide emotional support.

### Strengths and weaknesses of the study

One of the main strengths of this study is that it is based on a large sample of residents living in 12 deprived neighbourhoods with a response rate of 63%. This is noteworthy, as residents of deprived neighbourhoods are less likely to participate in health research [64, 65] and tend to be underrepresented in health profile surveys [19, 66]. It is the first study on perceived stress and health-risk behaviour carried out in a Danish context. Further, the study is unique in simultaneously examining four central health-risk behaviours (low intake of fruit and vegetable, smoking, high-risk alcohol intake, and physical inactivity) as well as the co-occurrence of health-risk behaviours.

Our study has some limitations. Non-response in DNHPS could affect the results regarding the level of perceived stress; if the most stressed people tend not to participate, the level of perceived stress may be underestimated. The respondents are considered to be representative of the residents of deprived neighbourhoods in Denmark, however, so we do not consider non-response to be a serious bias in relation to the observed associations. The cross-sectional data used for this study precluded the assessment of the directions of causations in the relationships between perceived stress and health-risk behaviour. The results should therefore be interpreted in the light of existing theories and research [27]. The association between perceived stress and health-risk behaviour could also operate in the reverse direction to that examined in the present study. As smoking, drinking, and physical inactivity can increase stress indicators, health-risk behaviour may also cause or worsen mental health problems [32]. A further limitation of our study is the measurement of perceived stress using an index based on four items. This might have certain drawbacks compared with Cohen’s 10-item PSS, which has been demonstrated to be a valid and reliable instrument on its own [38]. However, we were unable to include further items in the perceived stress index due to limitations in the length of the questionnaire used in the DNHPS*.* The four questions chosen were found to provide the best measure of perceived stress in deprived neighbourhoods. Another limitation is the use of two slightly different formulations of the second question in the measurement of perceived stress in the DNHPS and the DHMS. However, we do not consider that this has any major impact on the results. Furthermore, we do not know if some of the participants in the DNHPS also participated in the DHMS, as we do not have access to information about the respondents’ personal identification number in the DNHPS [7]. Different modes of data collection were used for the DNHPS (primarily telephone interviews) and the DHMS (paper or web-based self-administered questionnaires). In a narrative review by Bowling (2005), face-to-face and telephone interviews generally had a higher risk of social desirability bias than self-administered questionnaires [67]. This could cause social desirability response bias in regard to level of perceived stress and health-risk behaviour.

Further, our study was based on self-reported data, which may have led to information bias. People tend to overestimate their physical activity, which can be an indicator of social desirability bias [68].

## Conclusions

This study has shown that residents of deprived neighbourhoods have higher odds of perceived stress than the Danish population in general, indicating the need for health promotion interventions that are targeted at deprived neighbourhoods. Perceived stress was associated with health-risk behaviour in deprived neighbourhoods, although not for high-risk alcohol intake. The results of this study may provide support for the contention that residents of deprived neighbourhoods, in comparison with the general population, have higher odds of health-risk behaviour partly due to higher levels of perceived stress.

We suggest that health promotion interventions in deprived neighbourhoods would benefit from incorporating strategies to reduce perceived stress in regard to reducing health-risk behaviours. Longitudinal studies are needed to examine the causal association between perceived stress and health-risk behaviour over time. There is a continued need for a deeper understanding of the pathways by which perceived stress affects health-risk behaviour. This could help identify appropriate target areas for interventions aiming to change health-risk behaviour in deprived neighbourhoods.

## Additional files

Additional file 1: **Table S1.** Factor loadings for perceived stress index in deprived neighbourhoods and in the general population. (DOCX 15 kb)

Additional file 2: **Table S2.** Prevalence of perceived stress in deprived neighbourhoods and in general population. ORs with 95% CI for perceived stress in deprived neighbourhoods compared to general population. (DOCX 16 kb)

Additional file 3: **Table S3.** Prevalence of indicators of perceived stress in deprived neighbourhoods and in the general population. (DOCX 18 kb)

Additional file 4: **Table S4.** Prevalence of monthly disposable income in DKK in deprived neighbourhoods; **Table S5.** Prevalence of economic deprivation in deprived neighbourhoods: Had had to refrain from doing one or more of the listed activities within the last year for economic reasons; **Table S6.** Prevalence of strain in deprived neighbourhoods: Have been strained by some of the listed factors within the past year. (DOCX 16 kb)

## Abbreviations

CI: Confidence interval
DHMS: Danish Health and Morbidity Survey 2010
DNHPS: Deprived Neighbourhood Health Profile Survey
EFA: Exploratory factor analysis
OR: Odds ratio
PSS: Perceived Stress Scale
SES: Socioeconomic status
