# Supplementary material for: Associations between perceived stress, socioeconomic status, and health-risk behaviour in deprived neighbourhoods in Denmark: a cross-sectional study
Source: BMC Public Health. 2018 Feb 13;18:250. doi: 10.1186/s12889-018-5170-x (PMC5812195; doi:10.1186/s12889-018-5170-x)
Supplement: Supplementary file 1 — Table S1. Factor loadings for perceived stress index in deprived neighbourhoods and in the general population. (DOCX 15 kb) [file 12889_2018_5170_MOESM1_ESM.docx]

**Additional file 1**

Table S1. Factor loadings for perceived stress index in deprived neighbourhoods and in the general population

|  | Deprived  neighbourhoods and general population | Deprived  neighbourhoods | General population |
| --- | --- | --- | --- |
| Factor loadings |  | | |
| Felt difficulties were piling up so high that you could not overcome them | 0.85 | 0.84 | 0.85 |
| Felt nervous or stressed | 0.79 | 0.78 | 0.80 |
| Found that you could not cope with all the things that you had to do | 0.79 | 0.77 | 0.79 |
| Felt that you were on top of things | 0.70 | 0.71 | 0.70 |
| KMO^1^ | 0.78 | 0.77 | 0.78 |
| Variance explained | 2.46 | 2.42 | 2.49 |
| Cronbach's alpha^2^ | 0.79 | 0.78 | 0.79 |
| ^1^KMO: Overall Kaiser-Meier-Olkin measure of sampling adequacy. |  |  |  |
| ^2^The overall standardized Cronbach's coefficient Alpha. |  |  |  |
