# Supplementary material for: Associations between perceived stress, socioeconomic status, and health-risk behaviour in deprived neighbourhoods in Denmark: a cross-sectional study
Source: BMC Public Health. 2018 Feb 13;18:250. doi: 10.1186/s12889-018-5170-x (PMC5812195; doi:10.1186/s12889-018-5170-x)
Supplement: Supplementary file 2 — Table S2. Prevalence of perceived stress in deprived neighbourhoods and in general population. ORs with 95% CI for perceived stress in deprived neighbourhoods compared to general population. (DOCX 16 kb) [file 12889_2018_5170_MOESM2_ESM.docx]

**Additional file 2**

Table S2. Prevalence of perceived stress in deprived neighbourhoods and in general population. ORs with 95% CI for perceived stress in deprived neighbourhoods compared to general population

|  |  | |  | Unadjusted | |  | | Adjusted | | | | | | | | | | | |
| --- | --- | --- | --- | --- | --- | --- | --- | --- | --- | --- | --- | --- | --- | --- | --- | --- | --- | --- | --- |
|  | Deprived  neighbourhoods | General population | | Deprived  neighbourhoods | |  | | Deprived  neighbourhoods | | | | | | | | | | | |
|  | % | % | | OR | (95 % CI) |  | | OR^a^ | | (95 % CI) | | OR^b^ | | (95 % CI) | | OR^c^ | | (95 % CI) | |
| Perceived stress | 33.6 | 26.7 | | **1.39** | **(1.30-1.49)** |  | | **1.30** | | **(1.21-1.40)** | | **1.10** | | **(1.01-1.19)** | | **1.19** | | **(1.07-1.32)** | |
| **Bold values** indicate significant odds ratios. | |  | |  |  | |  | |  | |  | |  | |  | |  | |  |
| ^a^ Adjusted for sex, age and ethnic background. | | | |  |  | |  | |  | |  | |  | |  | |  | |  |
| ^b^ Adjusted for sex, age, ethnic background, educational level and cohabitation status. Analysis restricted to respondents aged 25 years or older. | | | | | | | | | | |  | |  | |  | |  | |  |
| ^c^ Adjusted for sex, age, ethnic background, educational level, cohabitation status and employment status. Analysis restricted to respondents aged 25-64 years and employed, unemployed, disability pensioners and other non-employed. | | | | | | | | | | | | | | | | | | |  |
|  |  |  |  |  |  |  |  |  |  |  |  |  |  |  |  |  |  |  |  |
