# Supplementary material for: Associations between perceived stress, socioeconomic status, and health-risk behaviour in deprived neighbourhoods in Denmark: a cross-sectional study
Source: BMC Public Health. 2018 Feb 13;18:250. doi: 10.1186/s12889-018-5170-x (PMC5812195; doi:10.1186/s12889-018-5170-x)
Supplement: Supplementary file 3 — Table S3. Prevalence of indicators of perceived stress in deprived neighbourhoods and in the general population. (DOCX 18 kb) [file 12889_2018_5170_MOESM3_ESM.docx]

**Additional file 3**

Table S3. Prevalence of indicators of perceived stress in deprived neighbourhoods and in the general population

|  | Deprived  neighbourhoods | | General population | |
| --- | --- | --- | --- | --- |
|  | % | n | % | n |
| Felt nervous or stressed |  |  |  |  |
| Very often | 6.6 | 335 | 2.6 | 4159 |
| Often | 11.1 | 569 | 9.3 | 4159 |
| Sometimes | 25.5 | 1302 | 33.7 | 12,685 |
| Almost never | 20.0 | 1020 | 34.2 | 4159 |
| Never | 35.9 | 1837 | 16.9 | 12,685 |
| Missing | 1.0 | 50 | 3.3 | 481 |
| Found that you could not cope with all the things that you had to do |  |  |  |  |
| Very often | 6.2 | 319 | 4.1 | 4159 |
| Often | 13.4 | 687 | 13.0 | 4159 |
| Sometimes | 25.6 | 1308 | 34.1 | 12,685 |
| Almost never | 21.4 | 1092 | 34.4 | 4159 |
| Never | 32.1 | 1642 | 12.2 | 12,019 |
| Missing | 1.3 | 65 | 2.2 | 312 |
| Felt that you were on top of things |  |  |  |  |
| Very often | 3.5 | 180 | 1.3 | 4159 |
| Often | 5.8 | 297 | 4.1 | 4159 |
| Sometimes | 16.5 | 844 | 14.7 | 12,685 |
| Almost never | 38.0 | 1943 | 48.0 | 4159 |
| Never | 34.4 | 1761 | 29.3 | 12,019 |
| Missing | 1.7 | 88 | 2.6 | 367 |
| Felt difficulties were piling up so high that you could not overcome them |  |  |  |  |
| Very often | 3.7 | 189 | 2.7 | 4159 |
| Often | 7.2 | 366 | 7.0 | 4159 |
| Sometimes | 18.1 | 924 | 23.5 | 12,685 |
| Almost never | 23.1 | 1180 | 39.7 | 4159 |
| Never | 46.7 | 2385 | 24.8 | 12,019 |
| Missing | 1.4 | 69 | 2.3 | 319 |
