# Supplementary material for: Associations between perceived stress, socioeconomic status, and health-risk behaviour in deprived neighbourhoods in Denmark: a cross-sectional study
Source: BMC Public Health. 2018 Feb 13;18:250. doi: 10.1186/s12889-018-5170-x (PMC5812195; doi:10.1186/s12889-018-5170-x)
Supplement: Supplementary file 4 — Table S4. Prevalence of monthly disposable income in DKK in deprived neighbourhoods; Table S5. Prevalence of economic deprivation in deprived neighbourhoods: Had had to refrain from doing one or more of the listed activities within the last year for economic reasons; Table S6. Prevalence of strain in deprived neighbourhoods: Have been strained by some of the listed factors within the past year. (DOCX 16 kb) [file 12889_2018_5170_MOESM4_ESM.docx]

**Additional file 4**

Table S4. Prevalence of monthly disposable income in DKK in deprived neighbourhoods

|  | % |
| --- | --- |
| 0-999 | 2.5 |
| 1000-1999 | 6.0 |
| 2000-2999 | 10.6 |
| 3000-3999 | 13.2 |
| 4000-4999 | 11.5 |
| 5000-5999 | 9.9 |
| 6000-6999 | 6.6 |
| 7000-7999 | 5.9 |
| 8000-8999 | 4.2 |
| 9000-9999 | 4.7 |
| >10,000 | 13.0 |
| Do not know | 10.3 |
| Missing | 1.7 |

Table A5. Prevalence of economic deprivation in deprived neighbourhoods: Had had to refrain from doing one or more of the listed activities within the last year for economic reasons

|  | % |
| --- | --- |
| Having leisure activities | 20.9 |
| Giving gifts for birthdays or other occasions | 16.4 |
| Going to the dentist | 22.3 |
| Buying essential medicines | 7.1 |
| Buying clothes or shoes for sport or exercise | 20.5 |
| Paying bills | 13.5 |
| Incidental expenses | 16.0 |

Table A6. Prevalence of strain in deprived neighbourhoods: Have been strained by some of the listed factors within the past year

|  | % |
| --- | --- |
| Your finances | 35.4 |
| Your housing situation | 18.2 |
| Your work situation | 27.0 |
| The relationship with your partner or your children | 15.2 |
| Illness of yourself | 39.0 |
| Illness from your partner, family or close friends | 33.2 |
| Other | 86.2 |
